# Supplementary material for: The prognostic efficacy of cell-free DNA hypermethylation in colorectal cancer
Source: Oncotarget. 2018 Jan 9;9(6):7010–22. doi: 10.18632/oncotarget.24097 (PMC5805532; doi:10.18632/oncotarget.24097)
Supplement: Supplementary file 1 [file oncotarget-09-7010-s001.pdf]

The prognostic efficacy of cell-free DNA hypermethylation in colorectal cancer

SUPPLEMENTARY TABLES

Supplementary Table 1: Gene names and known function

	Name	*Function*
ALX4	Aristaless-like homeobox 1	Skull and limb development
APC	Adenomatous polyposis	Cellular adhesion and β-catenin regulation
BMP3	Bone morphogenetic protein 3	Bone formation
BNC1	Basonuclin 1	Regulates proliferation and rRNA transcription
BRCA1	Breast cancer 1	DNA repair and gene transcription
CDKN2A	Cyclin dependent kinase inhibitor 2A	Cell-cycle regulation
HIC1	Hypermethylated in cancer 1	Gene transcription and cellular division
HLTF	Helicase like transcription factor	Gene transcription and cellular division
MGMT	O-6-methylguanine-DNA methyltransferase	DNA repair
MLH1	Mutl homolog 1	DNA repair
NDRG4	N-myc downstream-regulated gene 4	Cell-cycle progression and regulation of mitotic signals
NPTX2	Neuronal pentraxin 2	Synapse formation and non-apoptotic cell death
NEUROG1	Neurogenin 1	Neuronal differentiation
OSMR	Oncostatin M receptor	Cytokine receptor for cell signalling
PHACTR3	Phosphatase and actin regulator 3	Nuclear scaffolding of proliferating cells
PPENK	Preproenkephalin	Synaptic signalling
RARB	Retinoic acid receptor beta	Cellular signalling and retinoic acid binding
RASSF1A	Ras association domain family member 1A	Cell-cycle regulation and DNA repair
SDC2	Syndecan 2	Cellular adhesion, signalling, and cytoskeletal structure
SEPT9	Septin 9	Cell-cycle regulation and cytokinesis
SFRP1	Secreted frizzled related peptide 1	Modulator of Wnt signalling
SFRP2	Secreted frizzled related peptide 2	Modulator of Wnt signalling
SPG20	Spastic paraplegia 20	Regulates endosomal traffic and mitochondria function
SST	Somatostatin	Inhibitory hormone and regulator of endocrine system
TAC1	Tachykinin precursor 1	Neurotransmitting and vasodilation
THBD	Thrombomodulin	Inhibition of haemostasis
TFPI2	Tissue factor pathway inhibitor 2	Inhibition of haemostasis
VIM	Vimentin	Cell shape and integrity maintenance
WIF1	Wnt inhibitory factor 1	Cell fate regulation in oncogenesis and embryogenesis
WNT5A	Wnt family member 5A	Cell fate regulation in oncogenesis and embryogenesis

Note. Gene names and known function have been cross-matched with the RefSeq database (http://www.ncbi.nlm.nih.gov/refseq/).

Supplementary Table 2: Characteristics of gene specific primers and probes

See Supplementary File 1

Supplementary Table 3: Characteristics of the reference gene (*MEST*) primers and probes

		Outer primers		*Inner primers*		*Probes*	*Coordinates*	*Accession no.*
MEST U	(+)	GGTTTTAAAAG TTCGGTGTTTATT	(130)	TGTTGTGGTAA TTAGTATATTTT	(83)	CGCGAGTAGTTGTG TTTTGTTCGCG	130492052 – 130492181	NC_000007.14
	(−)	CCIAACAACTA CAACCACTCC		CAACCACTCCAA CATACACTACA				
MEST M	(+)	GCGATGGGTTT GTGCGC	(130)	CGACGTTTTAG TTTCGAGTC	(86)	CGATCGGTGGTC GGGTTCGATCG	130486225 – 130486355	NC_000007.14
	(−)	GAAAAACCGA TTACGCATACG		CGCTTCCTAAAA CCAAAAATTCTCG				

Note. The primer and probe sequences for the methylation specific polymerase chain reaction with the individual amplicon sizes represented as number of base pairs in brackets to the right of the outer/inner primers respectively. The NCBI accession number (no.) and the remapping coordinates of the amplified promoter regions are also presented. (+) Forward primer, (−) Reverse primer. (*U*) indicates the un-methylated version of *MEST*, (*M*) indicates the methylated version of *MEST*.
