# Supplementary material for: The prognostic efficacy of cell-free DNA hypermethylation in colorectal cancer
Source: Oncotarget. 2018 Jan 9;9(6):7010–22. doi: 10.18632/oncotarget.24097 (PMC5805532; doi:10.18632/oncotarget.24097)
Supplement: Supplementary file 2 [file oncotarget-09-7010-s002.docx]

| **Supplementary Table 2:** Characteristics of gene specific primers and probes | | | | | | | | |
| --- | --- | --- | --- | --- | --- | --- | --- | --- |
|  |  | *Outer primers* | | *Inner primers* | | *Probes* | *Coordinates* | *Accession no.* |
| *ALX4* | (+) | CGTTTTCGTTCGTCGTTTGC | (114) | TTTTTCGGAGGCGATAAGTTC | (85) | CGCGATTGTCGGTCGTCGTTAAAGTATCGCG | 44309869 – 44309983 | NC_000011.10 |
|  | ( ̶ ) | CGAACCCGACTCTTAACG |  | CGAACCCGACTCTTAACG |  |  |  |  |
| *APC* | (+) | ATTGCGGAGTGCGGGTC | (98) | AGTGCGGGTCGGGAAGC | (91) | CGCGATCGTTGGATGCGGAATCGCG | 112737725 – 112737823 | NC_00005.10 |
|  | ( ̶ ) | AATCGACGAACTCCCGACG |  | AATCGACGAACTCCCGACG |  |  |  |  |
| *BMP3* | (+) | TAGCGTTGGAGTGGAGAC | (114) | AGTGGAGACGGCGTTC | (96) | CGTCGAGCGGGTGAGGTTCGCGTATCGACG | 81031015 – 81031129 | NC_000004.12 |
|  | ( ̶ ) | CCAACCCCACTTACTACG |  | CTTACTACGCTAACCCAACG |  |  |  |  |
| *BNC1* | (+) | GTAGGTAGTTAGTTGGTTTTC | (102) | GTAGGTAGTTAGTTGGTTTTC | (83) | CGCGATCGTATTTACGGGAGTCGGAGTTTGATCGCG | 83284403 – 83284505 | NC_000015.10 |
|  | ( ̶ ) | GCGAAAATTCTCTATACG |  | GAAACAAACGACCCGAAACG |  |  |  |  |
| *BRCA1* | (+) | GTTTTTTGGTTTTCGTGGTAAC | (114) | TCGTGGTAACGGAAAAGCGCG | (83) | CGATCGGCGGCGTGAGCGTACG | 43125420 – 43125441 | NC_000017.11 |
|  | ( ̶ ) | AAACCCACAACCTATCCCCCG |  | CCGTCCAAAAAATCTCAACG |  |  |  |  |
| *CDKN2A* | (+) | TGTTCGGAGTTAATAGTATTTTTTTC | (133) | TTTCGAGTATTCGTTTATAGC | (111) | CGACGTGAAAGATATCGCGGTACTTCG | 21974925 – 21975058 | NC_000009.12 |
|  | ( ̶ ) | TTTCTTCCTCCGATACTAACG |  | TTTCTTCCTCCGATACTAACG |  |  |  |  |
| *HIC1* | (+) | GATATAACGTTTTTTTCGCGTC | (142) | TTCGGTTTTCGCGTTTTGTTC | (91) | CGCGACGGTCGTCGTTCGGGTTCGCG | 2056054 – 2056196 | NC_000017.11 |
|  | ( ̶ ) | ATACCCGCCCTAACGCCG |  | CGAAAACTATCAACCCTCG |  |  |  |  |
| *HLTF* | (+) | TTTGATTGTTGTAGAAGGAGAC | (121) | TAGAAGGAGACGGCGTC | (88) | CGCGATCGATTGGATTCGCGGCGAGATCGCG | 149086415 – 149086536 | NC_000003.12 |
|  | ( ̶ ) | CCGCAACCCTAAAACCG |  | AAAACAAATTCCGAACGCCG |  |  |  |  |
| *MGMT* | (+) | GATATGTTGGGATAGTTCGC | (129) | GATATGTTGGGATAGTTCGC | (119) | CGCGATCGTATCGTTTGCGATTTATCGCG | 129467213 – 129467342 | NC_000010.11 |
|  | ( ̶ ) | AAAAAACTCCGCACTTCCG |  | GCACTCTTCCGAAAACGAAACG |  |  |  |  |
| *MLH1* | (+) | TGGTTTTTTGGCGTTAAAATGTC | (123) | TGGTTTTTTGGCGTTAAAATGTC | (94) | CGCGATCTCGTCCAACCGCCGAATATCGCG | 36993529 – 36993652 | NC_000003.12 |
|  | ( ̶ ) | CATCTCTTTAATAACATTAACTAACCG |  | AAATAACTTCCCCCGCCG |  |  |  |  |
| *NDRG4* | (+) | GTATTTTAGTCGCGTAGAAGGC | (111) | TTACGCGCGAGGGGATC | (88) | CGCGATCGCGGTTCGTTCGGGATTAGTTGATCDCG | 58463524 – 58463635 | NC_000016.10 |
|  | ( ̶ ) | AATTTAACGAATATAAACGCTCG |  | AATTTAACGAATATAAACGCTCG |  |  |  |  |
| *NPTX2* | (+) | TTCGGTAGGTTAGAGTGTC | (94) | AGGTTAGAGTGTCGAGTAGC | (80) | CGCGATCGGTGCGGTTGTGAGACGGTGATCGCG | 98617274 – 98617368 | NC_000007.14 |
|  | ( ̶ ) | CTATCGTCTCGAAAATCGCG |  | TCGAAAATCGCGTACACCG |  |  |  |  |
| *NEUROG1* | (+) | GTTTATACGAGTTGATTTGATC | (92) | GTTGATTTGATCGTCGGC | (63) | CGCGATGCCCGACCGATCTCCTAAATCGCG | 135535860 – 135535952 | NC_000005.10 |
|  | ( ̶ ) | CTTAACCTAACCTCCTCG |  | CTCGCCTACAAAAACCACG |  |  |  |  |
| *OSMR* | (+) | TCGGTGTCGTTTTTGTTTAGC | (147) | TCGGTGTCGTTTTTGTTTAGC | (97) | CGCGATCTTCGGACGGCGTTCGGATCGCG | 38846295 – 38846442 | NC_000005.10 |
|  | ( ̶ ) | AAACGACTCGACACTATACG |  | CCCACCAAATAAACAAACCG |  |  |  |  |
| *PHACTR3* | (+) | GAGGTTTAGAGAGGTTAAGC | (98) | AGGTTAAGCGATTCGTTC | (82) | CGCGATCCAACAACGCTAAAACCGCTGATCGCG | 59577157 – 59577255 | NC_000020.11 |
|  | ( ̶ ) | AACGAAAAATATAACCGCCG |  | GCCGATTCCCCGCG |  |  |  |  |
| *PPENK* | (+) | CGCGTTATTTCGGGAATC | (133) | AGGCGATTTGAGTCGTTTTTAC | (112) | CGCGATCAAAGTTGTCGGTCGGGAGGATCGCG | 56446032 – 56446165 | NC_000008.11 |
|  | ( ̶ ) | GACAACCTCAACAAAAAATCG |  | GACAACCTCAACAAAAAATCG |  |  |  |  |
| *RARB* | (+) | AGTAGGGTTTGTTTGGGTATC | (127) | GGGTATCGTCGGGGTAGATTC | (113) | CGCGACGAATACGTTCCGAATCGCG | 25428388 – 25428515 | NC_000003.12 |
|  | ( ̶ ) | TCGACCAATCCAACCGAAACG |  | TCGACCAATCCAACCGAAACG |  |  |  |  |
| *RASSF1A* | (+) | GGGAGGCGTTGAAGTC | (115) | GGGAGGCGTTGAAGTC | (76) | CGCGATTCGTTCGGTTCGCTCGCG | 50340782 - 50340897 | NC_000003.12 |
|  | ( ̶ ) | AATAAACTCAAACTCCCCCG |  | GTACTTCGCTAACTTTAAACG |  |  |  |  |
| *SDC2* | (+) | GCGTAGGAGGAGGAAGC | (102) | GTTTCGAGTTCGAGTTTTC | (66) | CGCGATCCGTGAGTCGTAATCGTTGCGGTGATCGCG | 96494093 – 96494195 | NC_000008.11 |
|  | ( ̶ ) | CAACCCGCGCACACG |  | CGCACACGAATCCG |  |  |  |  |
| *SEPT9* | (+) | GTTTAGTATTTATTTTCGAAGTTC | (118) | GTTTAGTATTTATTTTCGAAGTTC | (93) | CGACGTATTTAGTTGCGCGTTGATCGACGTCG | 77373442 – 77373560 | NC_000017.11 |
|  | ( ̶ ) | GCCGAAAACGCTTCCTCG |  | CCTCCGCGCGACCCG |  |  |  |  |
| *SFRP1* | (+) | GAGGCGATTGGTTTTCGC | (149) | GGAGTTGATTGGTTGCGC | (90) | CGCGATGGTTCGGTCGTAATCGCG | 41309435 – 41309584 | NC_000008.11 |
|  | ( ̶ ) | CGCGACACTAACTCCG |  | CGCGACACTAACTCCG |  |  |  |  |
| *SFRP2* | (+) | GTTTTTCGGAGTTGCGCGC | (124) | GTTTTTCGGAGTTGCGCGC | (94) | CGACGTTTGTAGCGTTTCGTTCGCGTTGTTACGTCG | 153788922 – 153789046 | NC_000004.12 |
|  | ( ̶ ) | CTCTTCGCTAAATACGACTCG |  | CCGAAAAACTAACAACCGACG |  |  |  |  |
| *SPG20* | (+) | GAACGTTTTGGTTGTTAC | (142) | GAACGTTTTGGTTGTTAC | (123) | CGCGATCCGCTACGCTCGCCGAAATCGCG | 36346256 – 36346398 | NC_000013.11 |
|  | ( ̶ ) | CTCGAAAACTCCCTACG |  | ACCGCCGCGACACG |  |  |  |  |
| *SST* | (+) | AGAGTATATAAGTCGTTTTAGGAGC | (65) | GCGTCGAGATGTTGTTTTGTC | (81) | CGATCGACCAACGCGCACTAACGATCG | 187670234 – 187670315 | NC_000003.12 |
|  | ( ̶ ) | CCAAAACCAAAACGATAAACAACG |  | CCAAAACCAAAACGATAAACAACG |  |  |  |  |
| *TAC1* | (+) | TAAGGAGGTTGGGATAAATATC | (83) | ATCGTAAGGTATTGAGTAGGC | (64) | CGATCGATCCGAACGCGCTCTCGATCG | 97732034 – 97732126 | NC_000007.14 |
|  | ( ̶ ) | TCTCGATAACTACCGCCG |  | TCTCGATAACTACCGCCG |  |  |  |  |
| *THBD* | (+) | CGTCGTAGTAATTTATTTGTTATC | (119) | CGTCGTAGTAATTTATTTGTTATC | (99) | CGCGATCGTTCGGATTTCGGCGTTTGATCGCG | 23050377 – 23050496 | NC_000020.11 |
|  | ( ̶ ) | TAAACACGCACTAACCAACG |  | CCAAACCCCATCTCATCG |  |  |  |  |
| *TFPI2* | (+) | TATTTTTTAGGTTTCGTTTCGGC | (86) | TATTTTTTAGGTTTCGTTTCGGC | (72) | CGCGATCGTCGGTCGGACGTTCGTTGATCGCG | 93890745 – 93890831 | NC_000007.14 |
|  | ( ̶ ) | CGACTTTCTACTCCAAACG |  | AAACGACCCGAATACCCG |  |  |  |  |
| *VIM* | (+) | GAGGTTTTCGCGTTAGAGAC | (143) | ATATTTATCGCGTTTTCGTTC | (102) | CGACGTGTTCGCGTTATCGTCGTCGACGTCG | 17229296 – 17229439 | NC_000010.11 |
|  | ( ̶ ) | ACGAACCTAATAAACATAACTACG |  | ACGAACCTAATAAACATAACTACG |  |  |  |  |
| *WIF1* | (+) | CGACGCGTTTAGTC | (109) | GTTGAGGGAGTTGTAGC | (77) | CGCGATCGGTTGCGTAGGTGCGGGATCGCG | 65121211 – 65121320 | NC_000012.12 |
|  | ( ̶ ) | TACCGAAAAAACTCCTCG |  | TACCGAAAAAACTCCTCG |  |  |  |  |
| *WNT5A* | (+) | CGTGGAATAGTTGTTTGC | (152) | CGTGGAATAGTTGTTTGC | (134) | CGCGATCAACCTAATCGAAACGCAACTAAAGATCGCG | 55487159 – 55487311 | NC_000003.12 |
|  | ( ̶ ) | CGAACCTAAACTCCCG |  | TTAAAACAAAACTAAAATACG |  |  |  |  |
| Note. The primer and probe sequences for the methylation specific polymerase chain reaction with the individual amplicon sizes represented as number of base pairs in brackets to the right of the outer/inner primers respectively. The NCBI accession number (no.) and the remapping coordinates of the amplified promoter regions are also presented. (+) Forward primer, ( ̶ ) Reverse primer. | | | | | | | | |
